# Different Fgfs have distinct roles in regulating neurogenesis after spinal cord injury in zebrafish

**DOI:** 10.1186/s13064-018-0122-9

**Published:** 2018-11-17

**Authors:** Yona Goldshmit, Jean Kitty K. Y. Tang, Ashley L. Siegel, Phong D. Nguyen, Jan Kaslin, Peter D. Currie, Patricia R. Jusuf

**Affiliations:** 1 Australian Regenerative Medicine Institute, Monash University, Clayton, VIC 3800 Australia; 2 Steyer School of Health Professions, Sackler School of Medicine, Tel-Aviv University, P.O. Box 39040, 6997801 Tel Aviv, Israel; 3 School of Biosciences, University of Melbourne, Parkville, VIC 3010 Australia

**Keywords:** Motor neuron, Fgf2, Fgf3, Fgf8, Neural regeneration, Islet 1, C-met

## Abstract

**Background:**

Despite conserved developmental processes and organization of the vertebrate central nervous system, only some vertebrates including zebrafish can efficiently regenerate neural damage including after spinal cord injury. The mammalian spinal cord shows very limited regeneration and neurogenesis, resulting in permanent life-long functional impairment. Therefore, there is an urgent need to identify the cellular and molecular mechanisms that can drive efficient vertebrate neurogenesis following injury. A key pathway implicated in zebrafish neurogenesis is fibroblast growth factor signaling.

**Methods:**

In the present study we investigated the roles of distinct fibroblast growth factor members and their receptors in facilitating different aspects of neural development and regeneration at different timepoints following spinal cord injury. After spinal cord injury in adults and during larval development, loss and/or gain of Fgf signaling was combined with immunohistochemistry, in situ hybridization and transgenes marking motor neuron populations in in vivo zebrafish and in vitro mammalian PC12 cell culture models.

**Results:**

Fgf3 drives neurogenesis of Islet1 expressing motor neuron subtypes and mediate axonogenesis in cMet expressing motor neuron subtypes. We also demonstrate that the role of Fgf members are not necessarily simple recapitulating development. During development Fgf2, Fgf3 and Fgf8 mediate neurogenesis of Islet1 expressing neurons and neuronal sprouting of both, Islet1 and cMet expressing motor neurons. Strikingly in mammalian PC12 cells, all three Fgfs increased cell proliferation, however, only Fgf2 and to some extent Fgf8, but not Fgf3 facilitated neurite outgrowth.

**Conclusions:**

This study demonstrates differential Fgf member roles during neural development and adult regeneration, including in driving neural proliferation and neurite outgrowth of distinct spinal cord neuron populations, suggesting that factors including Fgf type, age of the organism, timing of expression, requirements for different neuronal populations could be tailored to best drive all of the required regenerative processes.

## Background

Spinal cord injury (SCI) triggers very limited regeneration in humans, resulting instead in irreversible damage which can lead to permanent paralysis. In contrast, non-mammalian vertebrates, such as fish and urodeles, regenerate damaged nerve cells in their spinal cords efficiently resulting in complete functional recovery even as adults [1–4]. Neurogenesis and neuronal survival, in particular of motor neurons is critical for improving functional recovery in mammals. However, mechanisms that influence neurogenesis in vertebrates after SCI are still not well understood and therapeutic strategies are therefore lacking.

Among the potential pro-regenerative neural factors, fibroblast growth factor (Fgf) signalling pathways have been shown influence angiogenesis, mitogenesis, cellular differentiation, cell migration and tissue-injury repair including in the developing and mature brain. In rodents and humans, 22 Fgf ligands [5] can be subdivided into subfamilies of intracellular (11–14) [6], hormone-like (15/21/23) and canonical Fgfs (1–10/16–20/22) [7–9]. Tissue-specific alternative splicing of the four receptors FgfR1–4 mRNAs [10] results in additional ligand – receptor combinations, and their distinct spatial-temporal expression patterns allow Fgf signalling to function in diverse biological processes. Cross-species comparisons revealed highly conserved conservation of Fgfs, with some mammalian Fgf homologues existing as two paralogues in teleosts including zebrafish, which have a total of 28 Fgfs [7]. Conserved developmental roles have been described in fish [11–13].

In the central nervous system, several Fgfs including 2, 3 and 8 are specifically expressed in the adult zebrafish brain including in progenitor zones [5, 14–17]. In mammals, Fgf2 is expressed in neurogenic zones, such as the cerebral cortex, colliculi, thalamus and olfactory bulb [18]. Fgf2 stimulates progenitor proliferation in adult mammalian hippocampal cell cultures [19–22], and when infused can increase neurogenesis in the mouse or rat dentate gyrus and sub-ependymal zone [23–26]. In adult mice, Fgf2 knockout decreases the number of dividing neural progenitors in the hippocampus and subventricular zone under normal or injury conditions [27, 28], and decreases the number of newborn neurons within the olfactory bulb [28] and motor cortex [29]. A single focal injection of FGF2 can prevent SCI-induced respiratory abnormalities, and improve recovery [30], by protecting choline acetyl transferase expressing ventral horn motor neurons from cell death. Fgf4, which is expressed ubiquitously in the adult zebrafish CNS [17], can promote neural progenitor proliferation and differentiation in adult neurospheres [31]. In adult zebrafish, Fgf3 and Fgf8 are highly expressed in neural progenitor niches such as the ventricular domains between the subpallium and olfactory bulb, midbrain and parvocellular preoptic nucleus (Fgf3 only) [17]. Fgf3 and Fgf8a are expressed in the ventral glial domain of the telencephalon. FgfRs in this area are expressed in more numerous cells resulting in broader downstream target molecules expression [14].

We and others have shown that aFGF (acidic Fgf1) or basic Fgf2 promote regeneration of axotomized spinal cord or dorsal root ganglion neurons in humans and animals after SCI [32–37], followed by functional motor behaviour improvement [33, 38]. In cortical neuron-glial cultures aFGF increased neuronal connections through AKT (protein kinase B) and ERK (extracellular signal-regulated kinase) activation, to effectively protect from oxygen glucose deprival induced neuronal damage [39]. In an in vivo cerebral ischemic rat model, aFGF stabilised within a fibrin glue reduces ischemic brain damage and microglial infiltration [39]. Thus, Fgf signalling shows highly conserved roles in the adult central nervous system including following injury.

Elucidating the role of different Fgfs during CNS regeneration allows us to target the appropriate Fgf ligand, and correct temporal window, to improve motor neuron regeneration. Two important markers labelling distinct motor neuron subpopulations are C-met and Islet1 [40]. C-met encodes the hepatocyte growth factor membrane receptor, which is expressed in quiescent muscle satellite stem cells as well as in the large cell bodied primary motor neurons (middle primary, rostral primary and caudal primary neurons) and the lateral line nerve [41–43]. Islet1 is transcription factors that is expressed in all cranial motor neurons, some cranial sensory neurons and postmitotic somatic motor neurons in the spinal cord. During development Islet1 expression in the smaller secondary motor neuron acts combinatorial with Lhx3 to promote motor neuron over V2 inteneuron differentiation. Islet1 is both required as well as sufficient for ectopic motor neuron upregulation [41–43]. These two markers thus label distinct motor neuron populations within the zebrafish spinal cord. Previously we demonstrated the role of Fgfs in gliogenesis and creation of glial bridges followed by axonal regeneration through the injured area [2]. Fgf3 and Fgf8 were demonstrated to be upregulated at the lesion site on radial glia cells and around motor neurons, therefore we hypothesised that these Fgfs may contribute to neurogenesis that was observed at the lesion site. By comparing the role of distinct Fgfs during regeneration and development of Islet1 and C-met motor neurons, as well as across species, we have started dissecting out differential roles of Fgfs that could be targeted selectively in novel therapeutic efforts.

## Methods

### Zebrafish strains

Adult fish (3–6 months old) and embryos of either sex were used from various strains. These include transgenic lines to visualise distinct cell populations: Tg(*gfap*:*EGFP*^*mi2001*^) labels radial glia cells across the central nervous system driven by the glial fibrillary acidic protein promoter [44], Tg(*Isl1*:*EGFP*^*rw0*^) labels secondary motor neurons in the spinal cord (additional to cranial motor and some sensory neurons) [45], Tg(*vsx1*:*GFP*) labels interneurons in the spinal cord driven by the visual homeobox 1 promoter [46], Tg(*met:GAL4; UAS:EGFP*)^ed6Tg^ [47] and Tg(*met:mcherry 2A KalTA4*)^pc24Tg^ use the C-met promoter to drive reporter expression in primary motorneurons, which represent a distinct population from neurons expressing Islet1 [48]. Additionally, two lines (obtained from Zebrafish International Resource Center) were used to manipulate Fgf signalling: Tg(*hsp70l:dn-fgfr1-EGFP*^*pd*^*)*^*1*^ in which heatshock induces expression of a dominant negative FgfR1 (Fgf signalling inhibition) [49], and *spry4−/−*^*fh117*^ mutants, which represent a gain of Fgf signalling function, as the key downstream negative regulator sprouty is missing. All experiments were conducted in accordance with Monash University guidelines and approved by the local ethics committee.

### Spinal cord lesion

Spinal cord lesioning and injections (intraperitoneal or lesion site) were performed as described previously [1, 2] in fully anesthetized fish. Fish were fully anaesthetized in buffered 0.033% tricaine methanesulfonate (MS-222) in fish tank water, until respiratory movements of the opercula stopped (3–5 min). Halfway between the dorsal fin and the operculum, corresponding to the eighth vertebra (approximately 5 mm caudal to the operculum) of the spinal cord, a longitudinal incision was made through the muscle layer, and the vertebral column was exposed by holding the muscle tissue aside. Then the vertebral column was cut completely with micro-scissors. The wound was sealed with a drop of 3 M Vetbond. Fish were recovered from the anesthesia, by flushing the gills of the fish in a tank of fresh fish water by gently pulling the fish through the water. Fish resumed breathing within a few seconds.

### Heat shock treatment for Fgf signaling inhibition

The dominant negative form of FgfR1 was induced by applying heat shock to Tg(*hsp70l:dn-fgfr1-EGFP*) transgenic or wildtype control animals. Animals were exposed to an increased temperature from 26 °C to 38 °C [49] and remained at 38 °C for 60 min, 4 h prior to spinal cord injury. Fish were exposed once daily to this heat shock regime and spinal cords collected at indicated time points.

### Bromo-deoxy-uridine injection

Intraperitoneal (IP) injections of 50 μl BrdU (2.5 mg/ml in PBS; Sigma, USA) were performed in fully anaesthetized fish immediately following SCI at 0, as well as at 2 and 4 days post lesion or in age-matched control non-lesioned fish.

### Fgf3 injections

Recombinant human Fgf3 (0.14 μg/injection/fish) [50] was injected IP into fully anaesthetized Tg(*Isl1:EGFP*) fish every second day starting immediately after SCI for 5 or 10 days. The central region of human Fgf3 shows 72% amino acid identity with zebrafish Fgf3 [51].

### Vivo morpholino injections

A single dose of 1 μl of 0.5 mM (5 μg/injection/fish) Fgf3 morpholino (5’CATTG TGGCATGGCGGGATGTCGGC3’) or vivo standard control morpholino (5’CCTCTTACCTCAGTTACAATTTATA3’) was injected into the lesion site immediately after spinal cord transection (Gene Tools, LLC, Oregon, USA). Fgf3 *vivo* morpholino injections in zebrafish larvae phenocopies the observed small otic vesicle seen in Fgf3 mutants.

### Tissue preparation

At different time points (3, 6 10 and 14 days) after SCI, fish were humanely killed by deep anaesthesia with buffered 0.2% MS-222. The brains and spinal cords were exposed and fixed for 2 h in 4% paraformaldehyde (PFA) in PBS (phosphate buffered saline) at room temperature. The brains and the spinal cords were subsequently dissected out and postfixed for a further 2–3 h in 4% PFA at room temperature followed by immersion in 30% sucrose in PBS overnight at 4 °C, before embedding in OCT (TissueTek). Spinal cords were cryostat sectioned at 20 μm thickness for immunohistochemistry or 30 μm thickness for in situ hybridization.

### Immunohistochemistry

Sections were labelled using standard immunohistochemical procedures to determine expression and localization of different proteins at the lesion site. Sections were post-fixed for 10 min in 4% PFA, followed by blocking solution (PBS-triton X containing 5% normal goat serum (Invitrogen, CA, USA)) for 1 h at room temperature. Antigen retrieval was performed by incubating the sections for 15 min in 2 M HCl prior to blocking for BrdU immunohistochemistry. Primary antibodies were diluted in blocking solution and sections were incubated overnight at 4 °C. After rinsing in PBS, sections were incubated for 2 h at room temperature with secondary antibodies diluted in blocking solution. Sections were mounted in Fluoromount (Dako, USA). Primary antibodies used were: mouse anti-NeuN (1:1000; Millipore); rabbit anti-pMAPK (mitogen-activated protein kinase 1:1000; Cell signalling); mouse anti-bromodeoxyuridine (1:400, Roche); rabbit anti-GFP (1:500; Invitrogen); mouse anti-β-tubulin (1:1000, Promega); rabbit anti-Ki67 (1:400, Thermo). Secondary antibodies used were: goat anti-rabbit or goat anti-mouse Alexa Fluor-488 or Alexa Fluor-594 (1:1000; Molecular Probes). Nuclei were visualised by staining with DAPI (4′,6-diamidino-2-phenylindole) (Sigma).

### Probe generation and in situ hybridization

In situ hybridization and probe generation was performed as previously described [14, 15]. Briefly, plasmids were linearized, transcribed and labelled, using T7 or SP6 polymerase (Roche) and a DIG RNA labelling mix (Roche). In situ hybridization was performed using standard procedures on 30 μm cryostat sections. Following staining, tissues were imaged using a Z1 AxioImager compound microscope. Prior to performing in situ hybridization, sections with cells expressing GFAP:EGFP or Isl1:EGFP were imaged allowing us to examine gene expression of the same glia or neuronal cells before and after in situ staining.

### Fgf exposure in larva

For Fgf exposure, Tg(*met:GAL4*; *UAS:EGFP*)^ed6Tg^ or Tg(*Isl1*:*GFP*^*rw0*^ */ met:mcherry 2A KalTA4*^*pc24Tg*^) double transgenic embryos were used at 24 h postfertilisation (hpf). Embryos were swum in 1.5 μg/ml Fgf3/8 or 2 diluted in embryo medium, or embryo medium alone (control) for 48 h. The embryo medium was replaced after the first 24 h.

### Microscopy

Following Fgf swimming exposure whole zebrafish embryos were mounted in 1% low melt agarose, covered by embryo medium containing 0.033% MS-222, and imaged using a 20X objective at the Zeiss LSM710 confocal microscope at 1 μm optical intervals. After imaging, embryos were fixed, sectioned and processed for GFAP immunoreactivity as described above. Sections were examined by brightfield or fluorescence microscopy using a Z1 AxioImager (Zeiss, Berlin, Germany) epifluorescence microscope. Photomicrographs (1300 × 1030 dpi) were obtained with various Plan-Neofluar objectives (Zeiss), and acquired as digital images using an AxioCam (Zeiss) digital camera with AxioVision software (v. 4.4; Zeiss). In order to confirm co-localization between different proteins, single optical plane sections of samples were acquired using the Apotome module and a 40X objective, using AxioVision software. All images were taken focused through the medial section containing the central canal identified in the DAPI channel without looking at the stained channels.

### PC12 rat pheochromocytoma cell culture

The PC12 cell line derived from rat pheochromocytoma (adrenal medulla) was kindly provided by A/Prof Julian Heng (Harry Perkins Institute of Medical Research). The PC12 cells were grown in Dulbecco’s modified Eagle’s medium supplemented with antibiotics, 10% heat inactivated fetal bovine serum and 10% horse serum (HS). Cells were incubated at 37 °C in 5% CO_2_ in air, and the medium was changed every 3–4 days. Cells were passaged when 90% confluent using PBS-EDTA (ethylenediaminetetraacetic acid). Cells were induced to differentiate by growing on polylysine-coated plates at a density of 5000 cells/well in a 24 well plate either in the presence or absence of 50 ng/ml hFgf2, hFgf3 or hFgf8 (R&D) without serum for 3 days. After 72 h, cells were fixed and immunostained using primary mouse anti-βIII-tubulin antibody (1:2000; Promega) and secondary anti-mouse Alexa – Fluor 564 antibody (1:1000; Molecular Probes) for quantification and length measurement of neurite outgrowth.

### Lysates preparation and immunoblot

For the p-MAPK signalling analysis, cells were plated at a density of 1 × 10^6^ cells/ 10 cm plate the day before the experiment. On the experimental day the medium was replaced with medium without serum and hFgf2/3/8 was added at different time points as indicated, and then lysed in lysis buffer (50 mM HEPES pH 7.5, 150 mM NaCl, 10% glycerol, 1% Triton X-100, 1 mM EDTA pH 8, 1 mM EGTA pH 8, 1.5 mM MgCl2, 200 μM Na3VO4, 150 nM aprotinin, 1 μM leupeptin and 500 μM 4-(2-aminoethyl) benzenesulfonyl fluoride hydrochloride). Protein concentration was determined using the Bradford assay (BioRad). An equal amount of protein was taken for each immunoblot. Equal amounts of protein from each sample were loaded and resolved by SDS-polyacrylamide gel electrophoresis through 10% gels. The gels were electrophoretically transferred to a nitrocellulose membrane. Membranes were blocked, blotted with the corresponding primary antibody (rabbit anti-pMAPK p44/p42 variant or MAPK 1:1000, Cell Signalling;) followed by secondary antibody linked to horseradish peroxidase. Immunoreactive bands were detected by chemiluminescence reaction.

### BrdU-positive cell quantification following adult SCI

The number of Isl1:EGFP only labelled cells or BrdU/Isl1:EGFP double labelled cells in the spinal cord sections were counted within a 200 μm^2^ grid located ~ 100–300 μm proximal to the lesion site from both sides of the lesion. This was done in images taken of every second serial longitudinal 20 μm thick section using the Z1 AxioImager (Zeiss, Berlin, Germany) with the ApoTome. Results were expressed as the mean ± SEM (*n* = 5 fish per group). Statistical significance determined using one-way ANOVA followed by multiple comparisons using the Tukey’s test.

### Islet1-positive cell and Islet1 / C-met neurite quantification in larva

The number of Isl1:EGFP labelled cells and the total neurite area of the Isl1:EGFP/ C-met:mCherry labelled cells were quantified from one side of the spinal cords in 3 dpf old larvae. For Islet1 positive cells counts, transverse 20 μm sections were taken from the area between the back fin and the anal fin, and single optical plane images were taken on a Z1 AxioImager with the ApoTome. Results were expressed as the mean ± SEM (*n* = 10 fish per group) and statistical significance determined using one-way ANOVA followed by multiple comparisons using the Tukey’s test.

For neuronal filament sprouting analysis, confocal stacks were loaded into Imaris (Bitplane) and neurite area was quantified using the FilamentTracer module. In the filament creation wizard, the seed points are first detected and thresholds were set to determine neurite starting points. The module then connected the seed points to create the spine and subsequent neurites. Neurite area was determined with manual thresholding (identical across image files) based on the actual fluorescence of the transgenic line. Results were expressed as the mean ± SEM (*n* = 8 fish per group) and statistical significance determined using one-way ANOVA followed by multiple comparisons using the Tukey’s test.

### C-met neurite quantification in adult

The number of C-met labelled neurites in Tg(*met:*GAL4*; UAS:EGFP)* fish was quantified at 350 μm distance distal and proximal to the lesion site 10 days following SCI. Results were expressed as the mean ± SEM (*n* = 7 fish per group; at least 27 sections of spinal cord from each group) and statistical significance was determined using the Student’s *t-*test.

### Proliferation and neurite outgrowth in PC12 cell line

The percentage of Ki67 labelled proliferating cells and the number of total cells extending β-tubulin labelled neurites was quantified and expressed as mean ± SEM of at least 10 fields / well in triplicate wells in at least *n* = 3 independent experiments. One-way ANOVA followed by multiple comparisons using the Tukey’s test was used to test for differences between experimental groups.

### NeuN positive cell counts

The number of NeuN positive cells were quantified and expressed as mean ± SEM. NeuN neuronal staining counted within a 200 μm^2^ grid located located from the centre of the lesion (*n* ≥ 7 per group). One-way ANOVA followed by multiple comparisons using the Tukey’s test was used to identify significant differences between groups in the experiments.

## Results

### Fgf signalling after spinal cord injury mediates neurogenesis of neurons at the lesion site

**Fig. 1:**
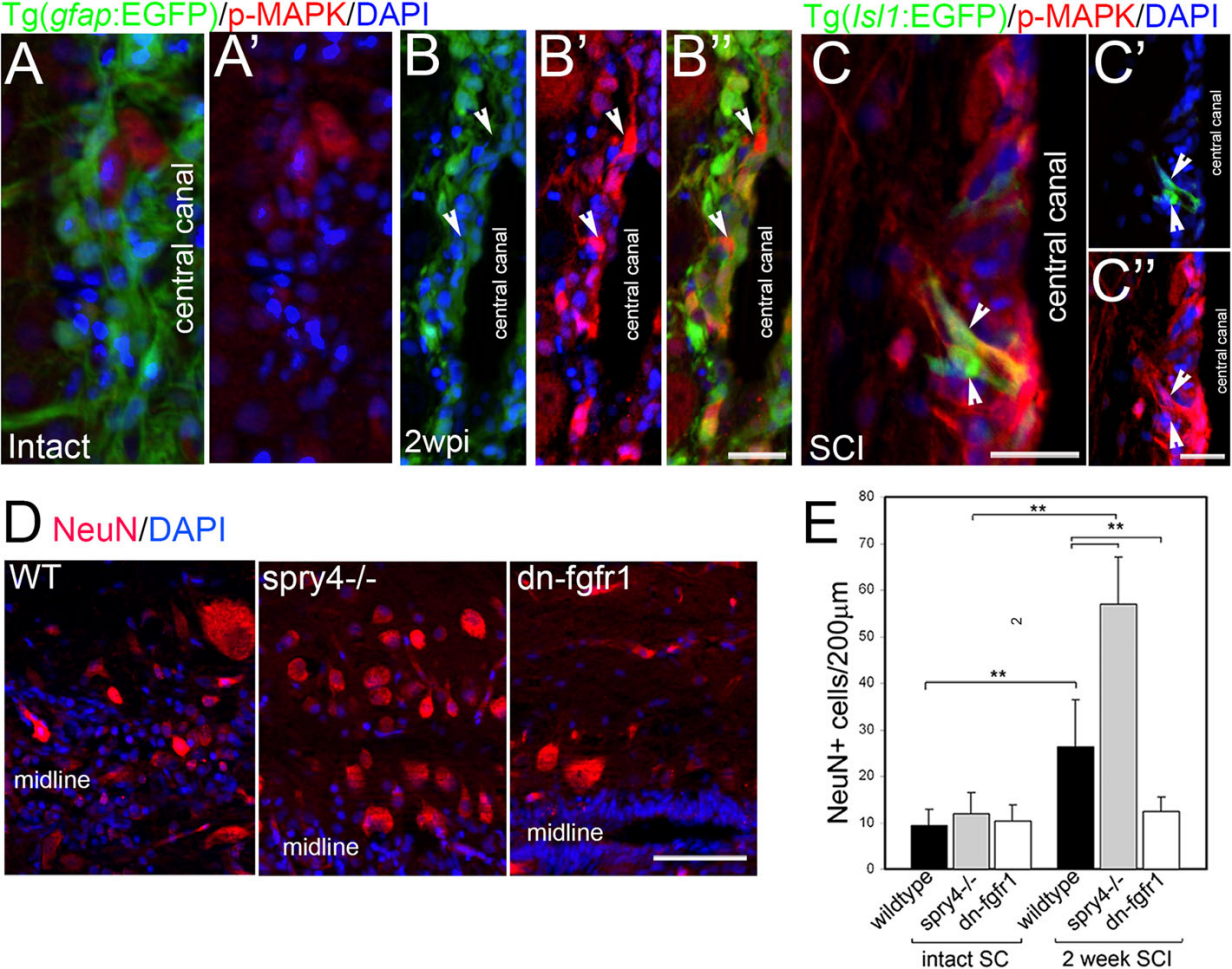
Fgf signalling increases neurogenesis after spinal cord injury. **a** Micrographs through intact non-injured adult zebrafish spinal cord show weak p-MAPK expression. **b** Micrographs through adult zebrafish spinal cord two weeks post injury (wpi) shows p-MAPK levels upregulated particularly in non-radial glia GFAP negative neurons at the central canal at the lesion site (arrows, B) (*n* = 5) some of which are Islet1 positive (**c**). **d**, **e** While Fgf signalling gain (*spry4−/−)* or loss (Tg(*hsp70l:dn-fgfr1-EGFP)* has no effect on NeuN+ neurons in intact spinal cord (SC), two weeks after injury, the significant increase in NeuN+ neurons in WT can be further increase with Fgf signaling gain and abolished with Fgf signaling loss. Graphs shows mean ± SEM, (*n* = 6 fish /group) ** *p* < 0.01. Scale bars in A, B and C are 25 μm, scale bar in D is 50 μm

In order to examine how and in which cells Fgf functions to influence neurogenesis following SCI, we examined the activation of p-MAPK (p44/42), a main downstream effector of the Fgf pathway at the lesion site at 2 weeks post-injury, when we observed highest generation of new neuronal cells in our previous study [2]. The Tg(*gfap*:*EGFP*)^mir2001^ zebrafish line, in which the glial fibrillary acidic protein promoter drives expression of the GFP reporter, was used to mark the ependymal radial glia cells of the spinal cord around the central canal, which are the resident stem cell population responsible for efficient neural regeneration post-SCI [2, 52]. In contrast to little p-MAPK expression in uninjured spinal cord (Fig. 1a, a’), p-MAPK activation can be observed 2 weeks post-SCI in the central canal at the lesion site including GFAP:EGFP negative cells, which could belong to a subpopulation of Olig2 positive glia cells, though at least some of these also had neuronal cell morphology (Fig. 1b-b”, arrowheads). While other tyrosine kinase receptors are also signalling through Ras-MAPK pathway [53], studies, including ours showed that Fgf is responsible for the full pattern of MAPK phosphorylation in drosophila, xenopus and zebrafish [2, 54–56]. This is has been demonstrated for Fgf3 and Fgf8 in zebrafish during subpallial region development in the brain [56]. We and others also previously showed that p-MAPK upregulation is blocked in dn-FGFR1 line and after using FgfR1 inhibitor, SU5402 [2, 56], indicating that during SCI neuronal regeneration in zebrafish p-MAPK is driven by Fgf signalling.

p-MAPK is upregulated after SCI shortly after injury (Fig. 1a). Examination in the Tg(*Isl1*:*EGFP*) zebrafish line, that drives GFP expression in motor neurons and ventral interneurons, revealed that increased p-MAPK at the lesion site co-labelled with Isl1:GFP positive neurons (Fig. 1b’, arrowhead). Thus, Fgf signalling is activated at the lesion site in newly regenerated neurons following SCI.

In order to directly assess whether Fgf is not only expressed in these new neurons, but directly influence their neurogenesis, the number of neurons at the lesion site was compared in Fgf loss and gain of function experiments. As a measure of neurogenesis, the number of immunolabelled NeuN positive neurons were compared between intact uninjured control spinal cords and spinal cords 2 weeks post-SCI (Fig. 1d, e). For loss of Fgf function, heat shock treatment was applied to Tg(*hsp70:dn-fgfr1*) zebrafish resulting in ubiquitous induction of the dominant negative FgfR1 receptor and efficient blockade of Fgf signalling [2, 14, 15, 49]. The loss of Fgf signalling had no significant effect in control intact spinal cords, but significantly blocked the increased number in NeuN labelled neurons observed in the wild type condition 2 weeks post-SCI. This suggests that Fgf signalling is indeed necessary for the increased neurogenesis at the lesion site. For gain of function, spinal cord injury was performed in the *spry4−/−* mutant zebrafish line. *Spry4* is a downstream target of Fgf signalling and functions as a potent feedback inhibitor of the Fgf pathway [57]. In the loss of function *spry4*−/− mutant the lack of Sprouty thus leads to an increase in Fgf signalling. While *spry4*−/− mutants had no change in NeuN labelled neuron numbers in the intact spinal cord, 2 weeks after SCI, the number of NeuN expressing neurons in *spry4−/−* mutants was significantly increased compared to intact and wild type post-SCI conditions (Fig. 1e). Thus, whilst increased Fgf signalling in the intact spinal cord has no effect on neurogenesis, its increase post-SCI is sufficient for further enhancing regenerative neurogenesis even in zebrafish. Taken together these results suggest that Fgf signalling after SCI in zebrafish is upregulated and acts via the MAPK pathway in newly generated neurons, being both necessary and sufficient for the observed regenerative neurogenesis.

### Fgf3 ligand mediating Islet1 neurogenesis

**Fig. 2:**
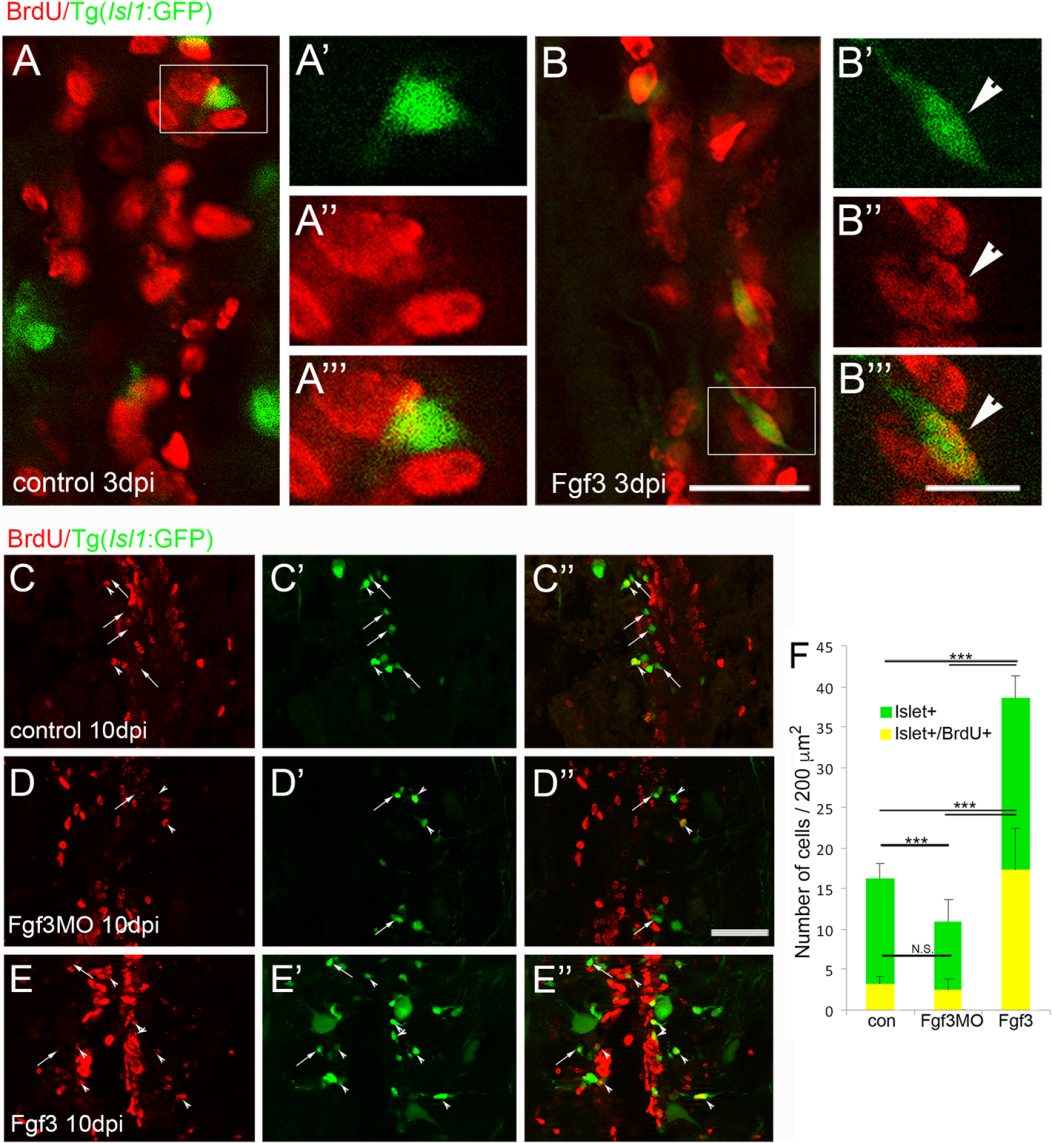
Fgf3 facilitates proliferation and neurogenesis of Islet1 motor neurons after spinal cord injury. **a**, **b** Three days post spinal cord injury (dpi), very few newly generated BrdU+ (red) cells express Islet1+ (green) motor neuron marker (a), unless treated with Fgf3 for three days (B, arrowhead). White box indicates region shown at higher magnification with individual and merged channels (A’ - A”’ and B’ – B”’). (C – F) Analysis of controls at 10 dpi shows that usually only a small proportion of newly generated BrdU+ cells usually become Islet1+ motor neurons (**c**, **f**). However, treatment with Fgf3 for three days facilitates both overall proliferation (increased number of BrdU+ cells) and specifically the proportion of newly generated cells that are becoming Islet1+ motor neurons (**e**, **f**), while overall Islet+ numbers, but not the newly generated BrdU+ cohort is significantly reduced when Fgf3 signalling is inhibited (**d**, **f**). Results in C show mean ± SEM, (*n* = 5 fish /group) *** *p* < 0.001; N.S.: not significant. Scale bar in B (for A and B) is 50 μm, scale bar in B”’ (for A’ – A”’ and B’ – B”’) is 10 μm and scale bar in F” (for D - F is 100 μm)

Specific upregulation of Fgf8 and Fgf3 mRNA levels after zebrafish SCI was previously demonstrated in radial glia and motor neurons cells respectively [58]. Thus, we examined whether intraperitoneal injection of Fgf3 and Fgf8 following SCI could mediate the observed regenerative neurogenesis. Quantification of the number of neurons in the Tg(*Isl1*:GFP) line at 5 and 10 days post-SCI revealed a significant increase in neurogenesis following Fgf3. Fgf8 did not show a significant increase in Islet-1 positive neurons after injury compared to control (12.23 + 6.12 SEM; 13.23 + 6.5 SEM respectively). We also examined Fgf3 mediated neurogenesis of interneurons after SCI using Tg(v*sx1*:GFP) zebrafish line, and did not observe any significant increase (19.28 + 5.3 SEM in control; 19.56 + 9.78 SEM in Fgf3 injected). This suggests that distinct Fgf ligands mediate regenerative neurogenesis of specific neuronal population. Quantification of BrdU incorporation as a marker for DNA synthesis and thus cell proliferation, revealed that newly generated Isl1:GFP/BrdU labelled neurons were already present 3 days after SCI in Fgf3 injected fish as opposed to PBS control injections, which did not result in any Isl1:GFP/BrdU double labelled cells at the lesion site at this time point (Fig. 2a, b).

At 10 days post-SCI, inhibition of Fgf3 using *vivo* morpholino injections directly into the lesion site significantly reduced the total number of Islet1+ cells (Fig. 2d, f). Additionally, Fgf3 *vivo* morpholino injections in zebrafish larvae phenocopies the observed small otic vesicle seen in Fgf3 mutants, though the results are relatively subtle. Consistent with this effect of Fgf3 inhibition, the opposite is observed when Fgf3 is upregulated using Fgf3 injections. After SCI, Fgf3 injection results in a significant increase both in the total number of Isl1:EGFP, but also in the number and proportion of Isl1:EGFP cells that are positive for BrdU labelling (Fig. 2e, f). Furthermore, the total number of Isl1:EGFP labelled small neurons (either BrdU positive or negative) 100–500 μm from lesion site centre is significantly increased following Fgf3 injection and decreased following Fgf3 inhibition compared to controls (Fig. 2c - f). In addition, Islet1 cells continue to be generated in the second week post injury as well similar to control fish. Therefore, neurogenesis following SCI is specifically mediated by Fgf3.

**Fig. 3:**
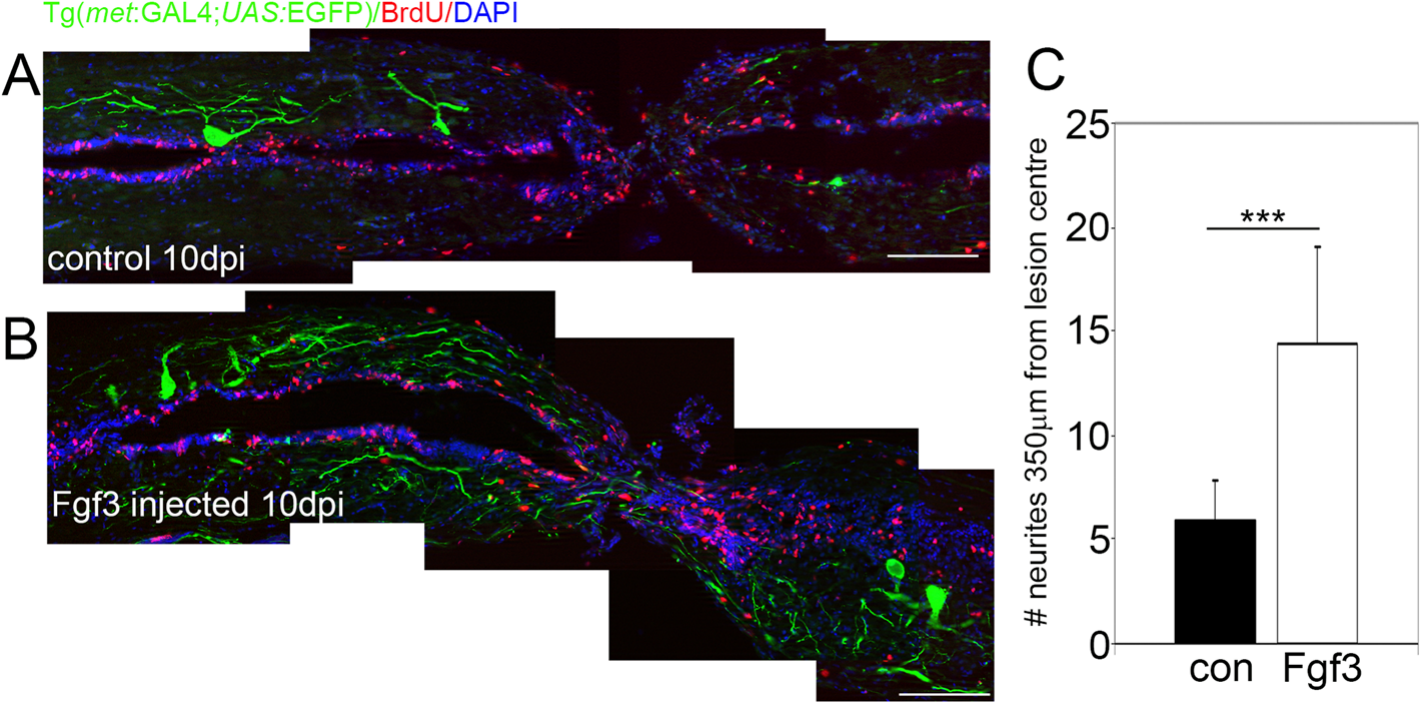
Fgf3 facilitates neurite sprouting of C-Met motor neurons after spinal cord injury. **a**, **b** Longitudinal sections through the spinal cord lesion site reveal that ten days post injury (dpi) Fgf3 treatment resulted in significantly more neurites at the lesion. Scale bars in A and B are 200 μm. **c** Quantitation of neurites up to 350 μm from lesion centre from both sides. Results are presented in C as mean ± SEM, (*n* = 7 fish /group) *** *p* < 0.001

In order to test whether Fgf3 specifically increases the number of Islet1 expressing motor neurons subpopulation, we also examined whether Fgf3 similarly mediates the proliferation of C-met expressing motor neurons post-SCI. There was no significant increase of C-met positive cells after Fgf3 treatment at the lesion site (data not shown). However, the number of C-met labelled neurites quantified post-SCI in Tg(*met*:*GAL4; UAS:EGFP*) was significantly increased at 350 μm from the lesion site centre 10 days post-SCI in Fgf3 injected compared to vehicle-controlled injected animals (Fig. 3 a-c), suggesting that particularly axonogenesis of the C-met neuronal subpopulation is mediated by Fgf3.

### Fgf receptor expression increases after SCI on neuronal cells

**Fig. 4:**
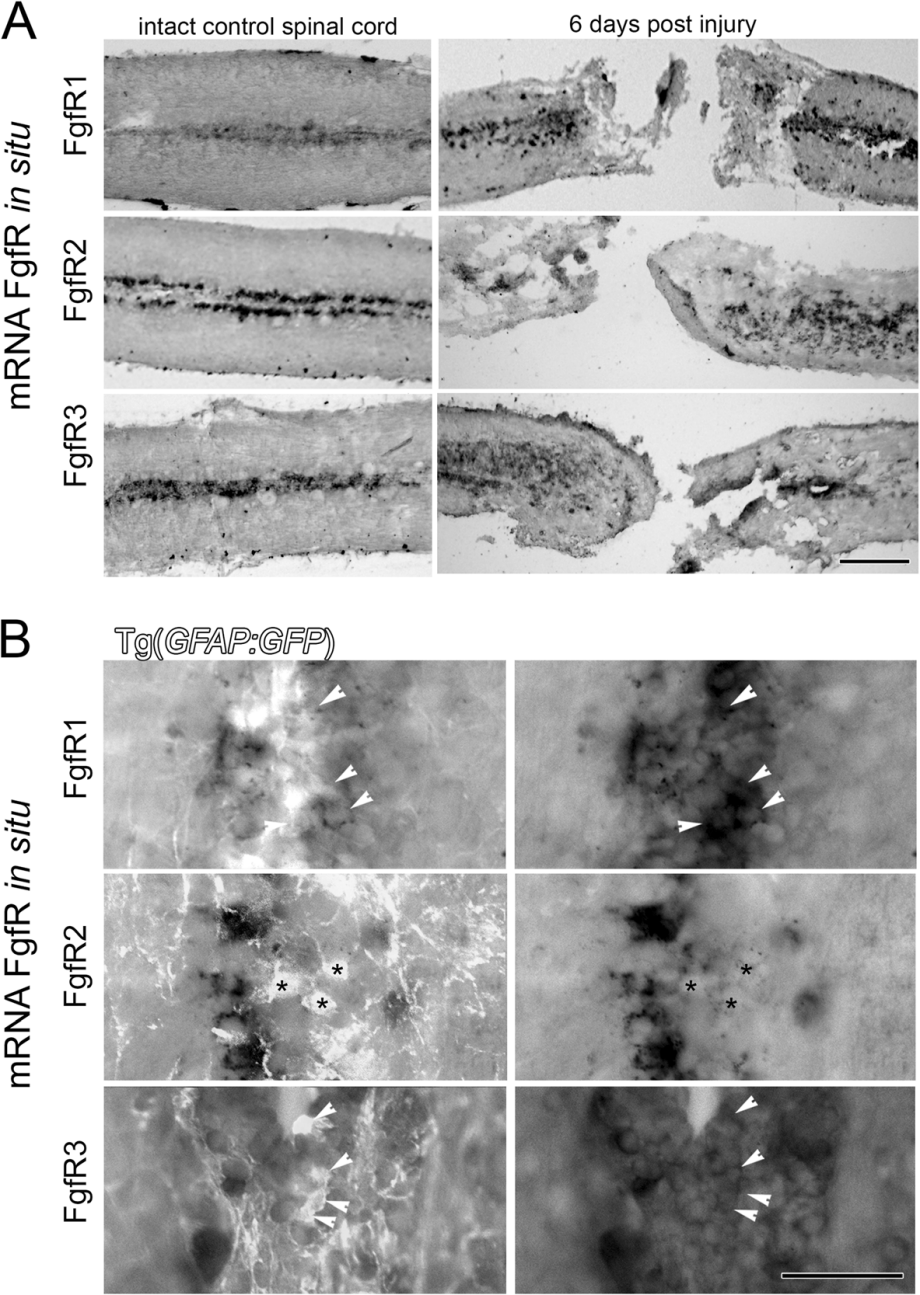
FgfRs expression around the central canal of the spinal cord on GFAP expressing radial glia. **a** Longitudinal sections show FgfR1–3 mRNA expression in cells at the central canal in intact uninjured spinal cord. At 6 days post injury (dpi) in situ hybridization shows an increase of the mRNA of all three FgfRs in these central canal cells particularly around the lesion site. **b** Sections from spinal cords in Tg(*gfap:*EGFP*)* transgenic fish show the location of GFAP+ ependymal radial glia cells and in situ hybridisation mRNA signal for FgfRs 1–3. At least some of the glia cells express varying levels of particularly FgfR1 and FgfR3 (arrowheads) with little overlap observed for FgfR2 (asterisks). Scale bar in A is 200 μm, Scale bar in B is 50 μm

**Fig. 5:**
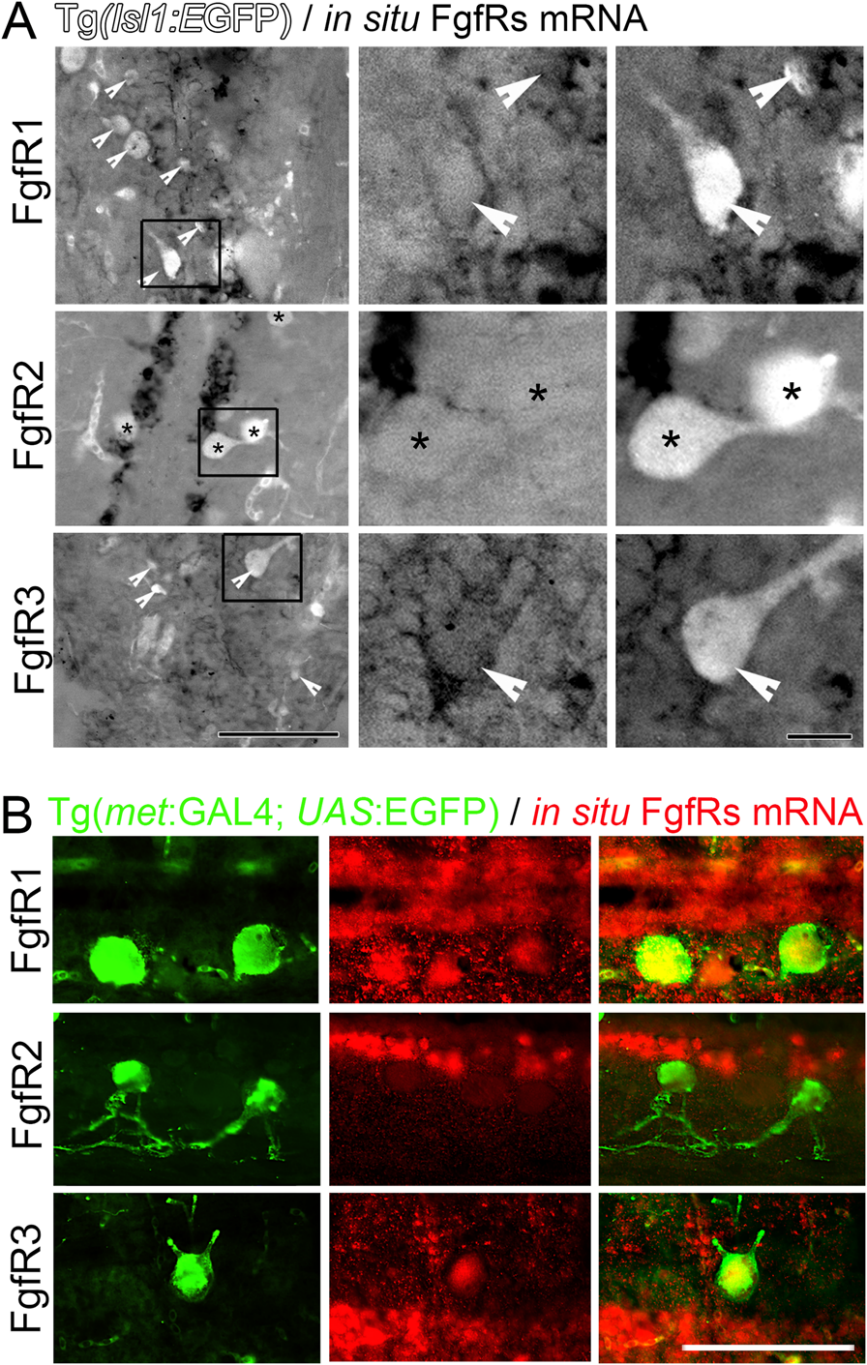
FgfRs expression in cells around the central canal and Islet1 and c-Met expressing motor neurons 6 days post spinal cord injury. **a** Sections from Tg(*Isl1:GFP)* transgenic fish showing the location of Islet1+ motor neurons compared to in situ hybridisation for FgfRs 1–3 mRNA. The middle and left panels are higher magnification insets of the boxes indicated in the left panels showing either FgfR signal alone (middle) or merged channels (right). At least some of the Islet1+ motor neurons express FgfR1 and 3 (arrowheads), but not FgfR2 (asterisks). **b** Sections from Tg(*met:GAL4; UAS:EGFP)* transgenic fish showing the location of c-Met+ motor neurons compared to in situ hybridisation for FgfRs 1–3 mRNA. Similarly as above, C-met neurons co-labelled with FgfR1 and 3, but not FgfR2 mRNA. The right panels show the merged C-met only (green - left panels) and in situ FgfR mRNA only (red - middle panels). Scale bar in A for left panels is 50 μm, and for middle and right panels is 10 μm. Scale bar in B is 50 μm

Understanding which receptors mediate the observed regenerative response allows us to better target these for specific and efficient regenerative strategies. Thus, in this study we examined Fgf receptor expression in the spinal cord. Cells around the central canal in the intact spinal cord express FgfR1, 2 and 3 (Fig. 4a). Following SCI, all of these Fgf receptors are upregulated as demonstrated at 6 dpi (Figs. 4 and 5). In order to assess specifically, which cell populations might be upregulating different Fgf receptor expression post-SCI, the expression of FgfR1, 2 and 3 was assessed in GFAP labelled radial glia and new Islet1 or c-Met-expressing neurons. It is important to note that an additional subpopulation of radial glia may expression low levels of GFAP and high levels of Olig2. While these progenitors usually give rise to oligodendrocytes, they also contribute motorneurons (BrdU+, Olig2:GFP +) following spinal cord injury [52].

By 6 days post-SCI, at least some of the FgfR1 and 3 mRNA (arrowheads), but not FgfR2 (asterisks) was co-localised with GFAP:EGFP expressing radial glia (Fig. 4b). For all three receptors there were additionally mRNA expressing cells that were not expressing the GFAP:GFP glia marker, but could belong to the Olig2+ glia subpopulation. Similarly, Isl1:EGFP positive neurons at the lesion site were among the cells that expressed FgfR1 and 3 mRNA (Fig. 5a, arrowheads). Although FgfR2 is strongly expressed at 6 dpi in the same area compared to control uninjured spinal cord, FgfR2 expression in Isl1:EGFP labelled neurons is low or absent (Fig. 5a, asterisks). Thus, FgfR1, 2 and 3 expression in radial glia of the intact spinal cord is increased post-SCI. In agreement, FgfR1 and FgfR3, but not FgfR2, are also upregulated on C-met expressing motor neurons after injury (Fig. 5b).

### Differential roles of Fgfs during developmental neurogenesis

**Fig. 6:**
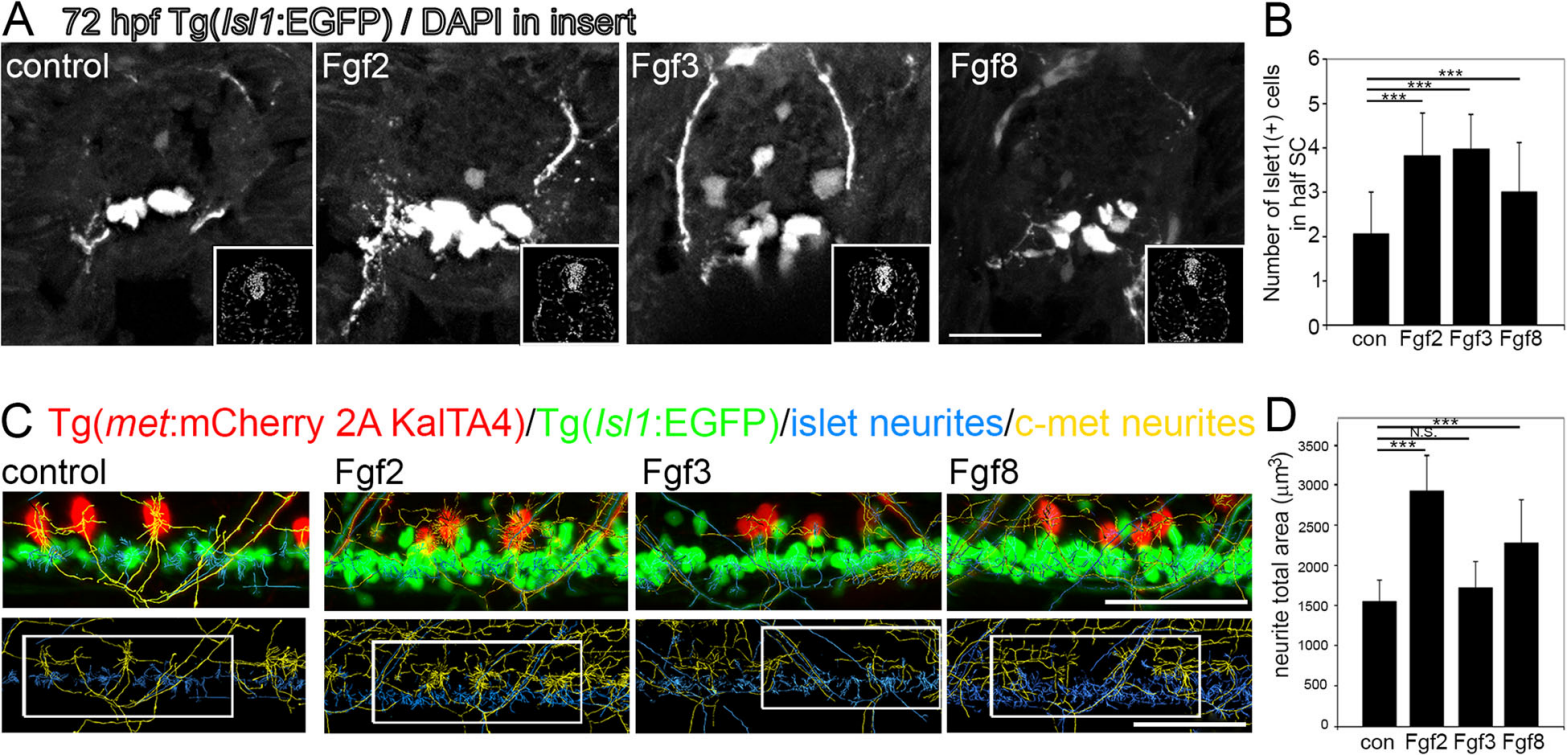
Fgfs mediate neurogenesis and neurite outgrowth during zebrafish development at three days postfertilisation. **a** Tranverse sections through Tg(*Isl1:GFP)* spinal cords after 48 h incubation in Fgf2, 3 or 8, showing Islet1+ motor neurons. Insets show DAPI nuclear labelling in lower right corner for each image. **b** Quantitation of half spinal cord in the sections at the level of the back-fin shows a significant increase in Islet1+ motor neurons following incubation in Fgf2, 3 or 8. Results are presented in B as mean ± SEM, (*n* = 10 fish/group)*** *p* < 0.001. **c** Representative images of longitudinal spinal cord images of double transgenic Tg(*Isl1:GFP)/* Tg(*c-met:mCherry)* fish incubated for 48 h in Fgf2, 3 or 8. Upper panel shows Islet1+ (green) and c-Met+ (red) transgenic label with Islet+ neuritis computationally annotated by Imaris software traced in blue and c-Met neurites computationally annotated by Imaris software traced in yellow. Lower panel shows an example of region of interest taken for analysis. **d** Quantitation of neurite total area of Islet1+ GFP and c-Met+ mCherry neurites reveals a significant increase in neurite outgrowth following Fgf2 and to a lesser extent Fgf8, but not Fgf3 incubation. Results are presented in D as mean ± SEM, (*n* = 8 fish/group) *** *p* < 0.001, N.S. = not significant. Scale bar in A is 50 μm, scale bar in C is 50 μm

In order to examine whether Fgf2, Fgf3 and Fgf8 have overlapping or distinct roles in mediating neurogenesis not only during regeneration, but also during normal neural development, 24 hpf embryos were swum in different Fgfs for 48 h. The number of neurons were quantified in cross sections of Tg(*Isl1*:*EGFP*) zebrafish spinal cords at the end of the dorsal fin, anterior to the anal vent. Compared to control, swimming in all three Fgfs significantly increased the number of Isl1:EGFP positive neurons (Fig. 6a, b). Using the double transgenic Tg(*Isl1:EGFP; met:mCherry 2A KalTA4*), Fgf2 and Fgf8 exposure during this early developmental stage was shown to also significantly increased the density of neurite sprouting from motor neurons (Isl1:EGFP and met:mCherry 2A KalTA4 labelled) as quantified from whole mount imaging (Fig. 6c, d). Thus, different Fgfs have mediate potentially distinct aspects of neurogenesis during development, with Fgf2 and Fgf8, but not Fgf3 being important for neurite sprouting as well as neurogenesis (number of neurons), which is enhanced by all three Fgfs. Furthermore, as Fgf8 did not enhance regenerative neurogenesis and Fgf3 did enhance neurite outgrowth during regeneration in adult zebrafish, the roles of distinct Fgfs and their respective ligands in regeneration may not necessarily recapitulate their role during developmental neurogenesis.

### Differential effects of distinct Fgfs in mammalian cells

**Fig. 7:**
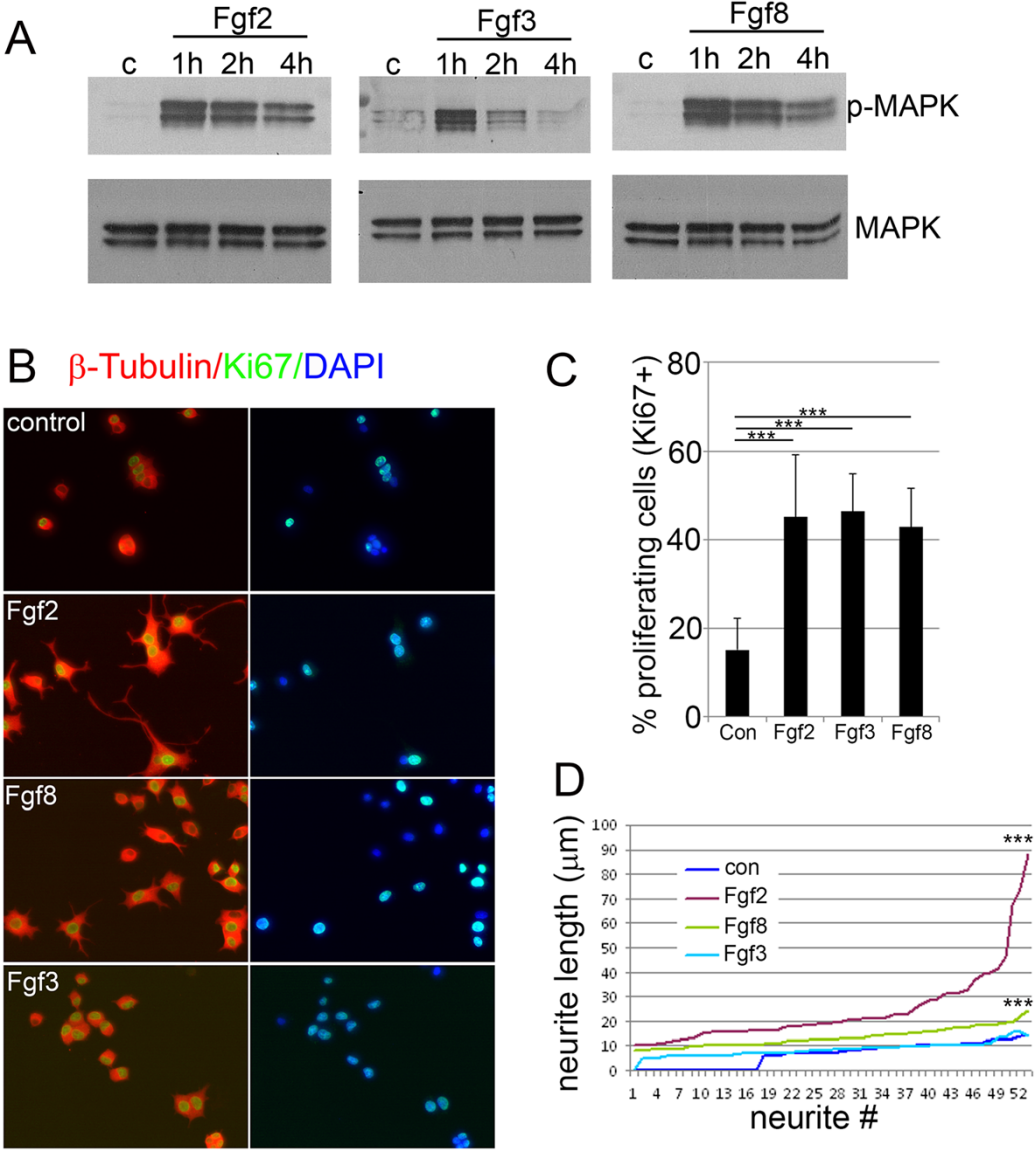
Fgfs increase neural proliferation and neurite outgrowth in mammalian PC12 cells. **a** Kinetics of MAPK activation (p-MAPK) shown in Western blots in control (**c**) or 1, 2 or 4 h following treatment with Fgf2, Fgf3 and Fgf8. Rapid activation of MAPK signaling occurs in response to all three Fgfs, but this activation is only transient in response to Fgf3 treatment as opposed to Fgf2 and Fgf8 treatment, which drives longer term activation. Total amount of MAPK is indicated by blotting the membrane with MAPK antibody. **b** Representative images of control versus Fgf2, 3 and 8 treated PC12 cells showing Ki67 immunostaining (green) labelling proliferation and b-tubulin immunostaining (red) labelling neurite morphology. **c** Quantitation of Ki67+ proliferative cells as a percentage of total DAPI nuclear cell counts shows significant increases in proliferation following treatment with any of the Fgfs. Results are presented as mean ± SEM, *** *p* < 0.001. **d** Neurite length sorted from shortest to longest show increased neurite length in cells treated with Fgf2 and Fgf8 but not Fgf3 compared to control. Neurite length *** *p* < 0.001

The rat pheochromacytoma cell line PC12 is a neuronal line which has been widely used as a mammalian model to study neuronal differentiation. These PC12 cells have previously been shown to extend neurites in response to both Fgf2 and Fgf8 [23, 59]. In PC12 cells all FGFR genes are apparently expressed, with FGFR1 being the most abundant [60]. Given the role we demonstrated for Fgf3 during adult spinal cord neural regeneration, the influence of Fgf3 on the mammalian PC12 cells was directly compared to the effect of Fgf2 and Fgf8, which have been demonstrated in the past as promoting neurite outgrowth in these cells [59, 61, 62]. Following incubation in either of those Fgfs (or none in the control condition) for 1, 2 or 4 h, Western blot analysis of the PC12 cells revealed a strong upregulation of p-MAPK following incubation in all three of the Fgfs compared to control. However, while incubation in Fgf2 and Fgf8 led to a sustained long-term activation of the p-MAPK pathway, Fgf3 exposure in contrast resulted in only a short-term activation of the p-MAPK pathway for less than 2 h (Fig. 7a). This difference in temporal p-MAPK activation pattern of Fgf signalling corresponds well with the observed long-term effect at 72 h after induction. While all three Fgfs increased cell proliferation as marked by Ki67+ immunolabelling (Fig. 7b, c), only Fgf2 and to a lesser extent Fgf8, but not Fgf3, increased neurite length quantified with β-tubulin staining (Fig. 7d).

These results are similar to those obtained during developmental neurogenesis in zebrafish and indeed the mammalian PC12 line may model a developmental rather than regenerative setting. Thus, the switch in Fgf roles between development and regeneration may be conserved across vertebrates.

## Discussion

We previously demonstrated that Fgf signalling mediates glia cell proliferation, differentiation and morphogenesis post-SCI in zebrafish [2]. We also observed neurogenesis of Islet1 positive cells at the lesion site, and therefore decided to examine Fgf candidates, Fgf8 and Fgf3, that were strongly upregulated at the lesion site, for this role. These activated glia are differentiating into neurons as well as glia cells, however, how the different Fgf ligands and their receptors contribute to distinct aspects of the regenerative neurogenesis is still unclear. For example, we did find GFAP+/c-Met+ double labelled cells during spinal cord development, therefore we believe that these GFAP+ radial glia cells that proliferate after SCI are responsible for neurogenesis. Additionally, other glia expression Olig2+ may also generate motor neurons [52, 63]. Our study demonstrated that Fgf signalling plays a pivotal role in neurogenesis after SCI in zebrafish, demonstrated by using *spry4−/−* mutant and dominant negative FgfR1 fish (gain and loss of Fgf signalling experiments respectively). Although Fgf signalling did not alter the number of neurons in the adult intact spinal cord, it significantly increased neurogenesis in *spry4−/−*, whilst decreasing neurogenesis in dn-FgfR1 post-SCI. This suggests that in injured tissue during the wound healing process, Fgf signalling is a critical mediator for efficient neurogenesis, which is a key step towards functional recovery of neural circuits. In the mammalian model of SCI, we could show that short term Fgf2 treatment increases neurogenesis after injury, consistent with data from other studies [33, 64]. Therefore the current study focused on examining the roles of Fgf2, Fgf3 and Fgf8 during neurogenesis and axonogenesis of two key motor neuron populations expressing Islet1 or c-Met [65–67]. These two gene promoters are activated in distinct populations of motor neurons within the zebrafish spinal cord, with C-met being expressed in quiescent muscle satellite stem cells and large cell bodied primary motor neurons (middle primary, rostral primary and caudal primary neurons) [47, 48] and Islet1 being expressed in cranial motor neurons and somatic motor neurons in the spinal cord. Other growth factors such as hepatocyte growth factor (HGF) have been shown to act as an axonal attractant and survival factor specifically for mammalian and avian motor neurons subpopulations [68–70] through Met [43], via intracellular signaling including mitogen activated protein kinase (MAPK) [71, 72], and thus we studied directly if different Fgf ligands could act in a similar way.

We now show that shortly after injury, Fgf3 treatment facilitates neurogenesis of Islet1 positive neurons, as demonstrated by positive BrdU labelling. We did not observe the same effect on neurogenesis after Fgf8 treatment or when assessing the effects of Fgf3 treatment on other spinal cord neuron populations such as Vsx1+ interneurons. The neurogenesis of Islet1 positive neurons is inhibited by Fgf3 *vivo* morpholino mediated knockdown. Injection of control *vivo* morpholino resulted in no observable change, suggesting that artefacts associated with the delivery are negligible. Similarly, no toxic effects were seen in embryos. Fgf3 injection is sufficient to increase axonogenesis in c-Met expressing motor neurons that were observed to cross the lesion site already at 10 days post-SCI. Similar injections of Fgf8 in contrast showed no significant difference (data not shown), but it is unclear whether there may be additional delivery or stability issues. Our results thus clearly demonstrate a specific role of Fgf3 during the regeneration of distinct motor neuron populations.

Because multiple receptors are expressed by the relevant cell populations investigated here, we directly assessed how their expression changed post-SCI. FgfR1–3 mRNA are expressed on glial cells in the uninjured adult spinal cord central canal. All of these three receptors are upregulated on these glia post-SCI. FgfR1 and FgfR3 were also upregulated specifically in Islet1 and c-Met expressing motor neurons. Although at this time point of 6 days post-SCI, FgfR2 mRNA was not detected in these neuronal cell populations, we previously demonstrated that FgfR2 protein was detected on large neurons at the injury site 2 weeks post-SCI. Therefore, Fgf3 may mediate neurogenesis through these different receptors possible acting through distinct receptors not only in different neuronal populations, but also at different timepoints following injury.

As regenerative processes often recapitulate at least some, but not necessarily all aspects of development [73], we directly compared the role of these Fgf ligands during developmental neurogenesis of the same motor neuron populations. During development immersion in all three Fgfs (Fgf2, Fgf3 and Fgf8) increases neurogenesis specifically of Islet1, but not c-Met expressing motor neurons. Additionally Fgf2 and Fgf8 significantly increased the total area occupied by neurites of both motor neuron populations, while Fgf3 in the double transgenic Tg(*Isl1*:*EGFP*) / Tg(*met:mCherry 2A KalTA4*) line did not. However, after SCI in the adult Tg(*met:mCherry 2A KalTA4*) transgenic line Fgf3 did increase the number of processes specifically at the lesion site. Thus, Fgf ligands and their receptors mediate neurogenesis and axonogenesis during development and after injury of the spinal cord. These results also demonstrate differences between developmental and regenerative roles of different Fgf ligands, suggesting an age-dependent functional switch or pathology versus development role within Fgf signaling pathways.

As a method to examine cross-species conservation of Fgf ligand function during vertebrate neurogenesis and neural differentiation, we performed experiments in the in vitro mammalian PC12 cell line. The neuronally related PC12 cell line expresses at least three of the FgfR at various levels, predominantly FgfR1, the stimulation of these cells with FGF ligand induces neuronal-like differentiation [60], we performed experiments in the in vitro mammalian PC12 cell line. We quantified the effects of Fgf2, Fgf3 and Fgf8 on proliferation and neurite outgrowth. These experiments revealed that all three Fgfs mediate MAPK pathway activation, either in the short term (1 h for Fgf3) or longer term (> 4 h for Fgf2 and Fgf8). Transient or prolonged MAPK activation has been demonstrated to mediate proliferation and neurite outgrowth respectively together with other growth factors [74]. Thus, we could show that in mammals, all three Fgfs induce cell proliferation, as quantified using proliferation marker Ki67, but only Fgf2 and Fgf8 induced neuronal differentiation and neurite sprouting. The similarities of these results describing the Fgf signalling role in this neuronal mammalian cell line compared to Fgf signaling role during zebrafish neural development could relate to the PC12 line representing a developmental rather than mature mammalian neurogenesis model, though this would need to be tested more thoroughly.

The role of Fgf2 during development in zebrafish correlates well with our mouse data demonstrating that Fgf2 injections mediate increase of Sox2 expressing cells at the lesion site two weeks post-SCI and in the long term increase neurogenesis of DCX positive cells, and double labelling of β-tubulin /BrdU cells at two months post-SCI [33]. The increase in neurite sprouting in zebrafish during development and after injury also correlates nicely with the increased axonal regeneration that we see in the mouse model at 2 and 4 months post-SCI following Fgf2 treatment.

## Conclusions

Together, our results represents an analysis of Fgf signaling in the adult spinal cord neurogenesis after injury in the zebrafish transection model. The widespread distribution of different Fgf members and their receptors in the central nervous reinforces the notion that Fgf signalling plays crucial roles in brain development and may also be critical after injury. It has become apparent that different ligands and receptors mediate distinct aspects of neurogenesis possibly at distinct times following neural injury. Furthermore, distinct roles may depend on the age of the animal and thus continued efforts in unravelling which ligand and which receptor will affect distinct cell types or processes at specific timepoint will contribute critical information towards designing tailored therapeutic intervention. Co-expression of p-MAPK and the neuronal marker Islet1, and the number of neurons that were born after injury in our Fgf gain and loss of function experiments further points to a prominent activation during events driving neurogenesis, such as neural injury.

## Abbreviations

BrdU: 5-bromo-2′-deoxyuridine
dpf: Days postfertilisation
dpi: Days post-injury
EDTA: Ethylenediaminetetraacetic acid
ERK/MAPK: Mitogen activated protein kinase
Fgf: Fibroblast growth factor
FgfR: Fibroblast growth factor receptor
IP: Intraperitoneal
PBS: Phosphate buffered saline
PFA: Paraformaldehyde
p-MAPK: Phosphorylated mitogen activated protein kinase
SCI: Spinal cord injury
SEM: Standard error of the mean
